# Clinical Characteristics and Complication Profiles of Patients Classified According to Cluster-Derived Diabetes Phenotypes

**DOI:** 10.3390/medicina62071396

**Published:** 2026-07-19

**Authors:** Doğan Aslan, Muammer Bilici, Sakin Tekin

**Affiliations:** 1 Internal Medicine Clinics, Hatay Defne State Hospital, Hatay 31000, Turkey; dogan.empires1990@gmail.com; 2 Department of Internal Medicine, Faculty of Medicine, Zonguldak Bulent Ecevit University, Zonguldak 67100, Turkey; 3 Department of Endocrinology and Metabolism, Faculty of Medicine, Zonguldak Bulent Ecevit University, Zonguldak 67100, Turkey; sakin.tekin@beun.edu.tr

**Keywords:** diabetes mellitus, cluster-derived phenotype, insulin resistance, complications, HbA1c follow-up

## Abstract

*Background and Objectives*: This retrospective study evaluated patients with diabetes classified according to cluster-derived diabetes phenotype groups and compared their baseline metabolic characteristics, documented complication profiles, and HbA1c course during follow-up. *Materials and Methods*: A total of 158 patients with type 1 or type 2 diabetes followed at Zonguldak Bülent Ecevit University Endocrinology Outpatient Clinic were included. Phenotype assignment was based on baseline domains corresponding to the Ahlqvist framework: age at diagnosis, BMI category, HbA1c, beta-cell function, insulin resistance, and autoantibody status. Complications were not used for phenotype assignment. Baseline characteristics, binary complication status, exploratory phenotype-contrast logistic regression, and longitudinal HbA1c data were evaluated. *Results*: The groups showed significant differences in age, age at diagnosis, HbA1c, obesity status, fasting glucose, C-peptide, fasting insulin, HOMA1-%B, HDL cholesterol, ALT, and eGFR. Hepatic steatosis/suspected NAFLD, retinopathy, nephropathy, polyneuropathy, and ketosis/ketoacidosis differed among groups when complications were analyzed as ever-positive versus negative. In exploratory phenotype-contrast logistic regression, Clusters 3–4 were associated with hepatic steatosis/suspected NAFLD, Cluster 2 with retinopathy and polyneuropathy, Cluster 3 with nephropathy, and Clusters 1–2 with ketosis/ketoacidosis; the contrast for coronary artery disease did not reach statistical significance. In a linear mixed-effects model for repeated HbA1c measurements, phenotype group was associated with HbA1c levels, whereas the time effect and group-by-time interaction were not statistically significant. *Conclusions*: Cluster-derived diabetes phenotype groups showed distinct baseline metabolic characteristics and different documented complication profiles. These findings should be interpreted as exploratory because of the retrospective design and incomplete follow-up data.

## 1. Introduction

Diabetes mellitus (DM) is a chronic, lifelong metabolic disease characterized by elevated circulating blood glucose levels, resulting from insufficient insulin secretion due to pancreatic β-cell destruction, impaired cellular response to insulin, or a combination of both mechanisms. The prevalence of DM is increasing worldwide and is expected to reach 592 million by 2035, leading to substantial human, economic, and social costs [1].

DM imposes a considerable burden on society through reduced productivity, decreased quality of life, increased healthcare expenditures, and premature mortality. The global cost of DM has been reported to be US$1.31 trillion, corresponding to 1.8% of the global gross domestic product. Indirect costs account for 34.7% of this total burden [2]. Furthermore, DM significantly increases mortality risk, with approximately one in twelve all-cause deaths being attributable to DM [3,4]. Despite the availability of effective treatment options, individuals with DM have a higher incidence of serious microvascular and macrovascular complications, including stroke, acute coronary events, blindness, amputation, kidney disease, and heart failure, as well as higher premature mortality compared with the general population [5].

The management of DM is challenging because patients differ in disease severity, sociodemographic characteristics, and clinical factors. Variables such as glycated hemoglobin (HbA1c), insulin sensitivity, body composition, and disease duration may contribute to clinical heterogeneity in diabetes. Therefore, classifying DM solely as type 1 or type 2 may be insufficient to fully describe the heterogeneity observed in clinical practice [6].

In this context, Ahlqvist et al. proposed a novel classification of adult-onset DM using K-means cluster analysis, identifying five subgroups: severe autoimmune diabetes (SAID), severe insulin-deficient diabetes (SIDD), severe insulin-resistant diabetes (SIRD), mild obesity-related diabetes (MOD), and mild age-related diabetes (MARD) [7]. This classification is based on six measurements commonly obtained in clinical practice: body mass index (BMI), age at DM diagnosis, HbA1c level, beta-cell function, insulin resistance, and the presence of DM-associated autoantibodies. These subgroups have been reported to differ in clinical characteristics and complication patterns. Accordingly, there is growing interest in identifying more homogeneous subgroups of patients with DM to better describe disease heterogeneity [7,8].

The Ahlqvist/Zaharia phenotype framework provides a pragmatic way to describe diabetes heterogeneity using clinically available baseline variables. In the present study, this framework was used as a reference for retrospective phenotype assignment rather than as a newly derived unsupervised clustering solution.

This study aimed to evaluate patients with diabetes followed at the Endocrinology Outpatient Clinic of Bülent Ecevit University Research and Application Hospital according to previously described cluster-derived diabetes phenotype groups and to compare their baseline metabolic characteristics, documented complication profiles, and HbA1c course during follow-up.

## 2. Materials and Methods

This retrospective study was conducted at the Endocrinology Outpatient Clinic of Zonguldak Bülent Ecevit University. This retrospective study included adult patients with type 1 or type 2 diabetes mellitus who were followed at the Endocrinology Outpatient Clinic of Zonguldak Bülent Ecevit University and had accessible diagnostic and follow-up data in the electronic health records. The study population consisted of patients with type 1 or type 2 diabetes mellitus registered in the Mia-Med patient follow-up system of our hospital. Clinical, laboratory, and imaging data were obtained retrospectively from the Mia-Med electronic health record system. For patients diagnosed at external centers and subsequently followed in our outpatient clinic, diagnostic data were reviewed through the national e-Nabız system with patient permission. Patients whose diagnostic data could not be accessed or whose required permissions could not be obtained were excluded.

Phenotype assignment was based on baseline domains corresponding to the Ahlqvist/Zaharia framework: age at diabetes diagnosis, body mass index (BMI) category, HbA1c at diagnosis, beta-cell function assessed by C-peptide and calculated HOMA1-%B, insulin resistance assessed by recalculated HOMA-IR, and diabetes-associated autoantibody status. Anti-GAD, islet cell antibody, and anti-insulin antibody results were used for autoantibody assessment. No new unsupervised clustering algorithm was performed in the present dataset; instead, patients were retrospectively assigned to previously described cluster-derived phenotype groups. Outcome variables were not used in phenotype assignment.

Laboratory parameters included fasting plasma glucose, HbA1c, fasting insulin, estimated glomerular filtration rate (eGFR), alanine aminotransferase, aspartate aminotransferase, spot urine albumin/creatinine ratio, total cholesterol, LDL cholesterol, HDL cholesterol, triglycerides, islet cell antibody, and anti-insulin antibody. Clinical parameters included height, weight, BMI, body composition distribution, and histories of diabetic retinopathy, nephropathy, diabetic polyneuropathy, coronary artery disease, and cerebrovascular events. Abdominal imaging reports were reviewed for hepatic steatosis/suspected NAFLD. The term NASH was not used unless explicitly documented by histological or validated noninvasive criteria.

After phenotype assignment, patients were evaluated from the date of diabetes diagnosis to the most recent available follow-up. During follow-up, blood glucose and HbA1c measurements, diabetes prescriptions, dyslipidemia data, and consultation and examination results related to diabetes complications were reviewed. HbA1c measurements were categorized according to predefined time intervals after diabetes diagnosis: 0–2 years, 2–4 years, 4–6 years, 6–8 years, and 8–10+ years. The final interval was open-ended and included available observations beyond 10 years when present. Retinopathy, nephropathy, neuropathy, coronary artery disease, cerebrovascular events, ketosis/ketoacidosis, and hepatic steatosis/suspected NAFLD were evaluated as outcomes and were not used for phenotype assignment. For the main complication analyses, time-coded outcome variables were collapsed into binary categories: those never documented versus those documented at any time during follow-up.

The local laboratory reference range for C-peptide was 1.1–4.4 ng/mL, and values below 1.1 ng/mL were considered consistent with reduced insulin secretion. HbA1c at diagnosis was used as a baseline phenotype variable, and serial HbA1c measurements were used to evaluate glycemic course during follow-up. HOMA-IR was recalculated as fasting glucose × fasting insulin/405. HOMA1-%B was calculated using the classical formula 360 × fasting insulin/(fasting glucose − 63) when both values were available; this value should not be interpreted as Oxford HOMA2-%B. BMI ≥ 30 kg/m^2^ was accepted as obesity. Baseline phenotype variables were evaluated together when assigning patients to phenotype groups.

For phenotype assignment, autoantibody positivity, early age at diagnosis, low BMI, insulin deficiency, and poor metabolic control were considered determinants of the severe autoimmune diabetes group. Low beta-cell function, high fasting plasma glucose, and high HbA1c were considered determinants of the severe insulin-deficient diabetes group. High BMI and HOMA-IR were associated with the severe insulin-resistant diabetes group, whereas patients with obesity but milder insulin resistance were classified into the mild obesity-related diabetes group. Patients diagnosed at older ages who were GADA-negative and had a milder clinical course were classified into the mild age-related diabetes group.

To avoid circularity, complication-related variables, including retinopathy, nephropathy, coronary artery disease, cerebrovascular events, ketosis/ketoacidosis, and hepatic steatosis/suspected NAFLD, were not used to define phenotype groups. These variables were analyzed only as follow-up outcomes. Clinical course and documented complication profiles were then compared among the phenotype groups.

Patients younger than 18 years of age, those with incomplete examinations or follow-up data, chronic obstructive pulmonary disease, Child-Pugh B or C cirrhosis before diabetes diagnosis, pregnancy, heart failure diagnosed before diabetes, uncontrolled or non-remitting malignancy, and secondary diabetes were excluded from the study.

The study was approved by the Ethics Committee of Zonguldak Bülent Ecevit University Faculty of Medicine (approval date: 11 December 2024, decision no: 2024/22). Study data were used only for scientific research purposes, and no personally identifiable patient information was included in the research report or shared with third parties or institutions. Continuous variables are presented as median (interquartile range), and categorical variables are presented as *n*/*N* (%). Continuous variables were compared using Kruskal–Wallis tests, and categorical variables were compared using chi-square tests. Time-coded complication variables were collapsed into binary variables for the main outcome analyses. Exploratory phenotype-contrast logistic regression analyses were performed for selected binary outcomes. For each outcome, the phenotype group or clinically related phenotype category with the highest observed frequency was compared with the remaining groups. These models were adjusted for current age, sex, and diabetes-duration category. Baseline HbA1c was not included in these focused contrast models because HbA1c at diagnosis was one of the phenotype-assignment variables, and its inclusion could introduce overadjustment or collinearity. Because these analyses were exploratory and post hoc, no formal correction for multiple testing was applied, and *p* values should be interpreted cautiously. Longitudinal HbA1c measurements were evaluated using a linear mixed-effects model with patient-level random intercepts and fixed effects for phenotype group, follow-up interval, and the group-by-time interaction.

## 3. Results

### Distribution of the Study Population Across Phenotype Groups

**Figure 1:**
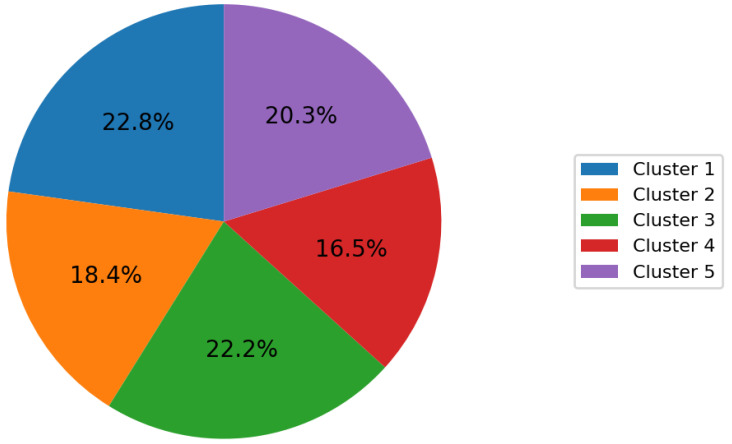
Distribution of patients according to phenotype groups.

A total of 158 patients were included in the study. Among them, 36 patients (22.8%) were classified as Cluster 1, 29 patients (18.4%) as Cluster 2, 35 patients (22.2%) as Cluster 3, 26 patients (16.5%) as Cluster 4, and 32 patients (20.3%) as Cluster 5. The distribution of patients across phenotype groups was generally balanced (Figure 1). Baseline characteristics are shown in Table 1. The groups differed significantly in age, sex distribution, age at diagnosis, baseline HbA1c, obesity status, autoantibody positivity, fasting glucose, C-peptide, fasting insulin, HOMA1-%B, HDL cholesterol, ALT, and eGFR. HOMA-IR and triglyceride levels were numerically higher in insulin resistance-related groups but did not significantly differ across groups in the continuous-variable analysis.

For complication analyses, time-coded outcomes were collapsed into binary variables indicating whether each outcome was ever documented during follow-up. Hepatic steatosis/suspected NAFLD differed significantly among groups and was most frequent in Clusters 3 and 4. Diabetic nephropathy, retinopathy, polyneuropathy, and ketosis/ketoacidosis also differed significantly among groups (Table 2).

Microvascular complications showed different distributions across phenotype groups. Diabetic nephropathy was most frequent in Cluster 3, whereas retinopathy and polyneuropathy were most frequent in Cluster 2. These results were based on binary ever-positive versus negative classification and are summarized in Table 2.

Ketosis/ketoacidosis was more frequent in Clusters 1 and 2 and least frequent in Clusters 4 and 5 when evaluated as ever documented during follow-up (Table 2).

When macrovascular complications were evaluated using binary outcome status, coronary artery disease did not differ significantly among groups (*p* = 0.070), and cerebrovascular events were rare (*p* = 0.208) (Table 2).

Exploratory phenotype-contrast logistic regression analyses were fitted for selected binary outcomes (Table 3). Clusters 3–4 were strongly associated with hepatic steatosis/suspected NAFLD compared with the remaining groups (adjusted OR 39.84, 95% CI 11.13–142.67; *p* < 0.001). Cluster 2 was associated with higher odds of diabetic retinopathy (adjusted OR 4.51, 95% CI 1.84–11.07; *p* = 0.001) and diabetic polyneuropathy (adjusted OR 3.52, 95% CI 1.41–8.77; *p* = 0.007), whereas Cluster 3 was associated with higher odds of diabetic nephropathy (adjusted OR 7.67, 95% CI 3.15–18.70; *p* < 0.001). Ketosis/ketoacidosis was more frequent in the insulin-deficient phenotype contrast, defined as Clusters 1–2 versus the remaining groups (adjusted OR 4.15, 95% CI 1.94–8.88; *p* < 0.001). In contrast, the association between Clusters 2–3 and coronary artery disease did not reach statistical significance (adjusted OR 2.08, 95% CI 0.92–4.71; *p* = 0.078). These analyses were considered exploratory because the contrasts were selected according to observed complication patterns and clinical phenotype structure.

**Figure 2:**
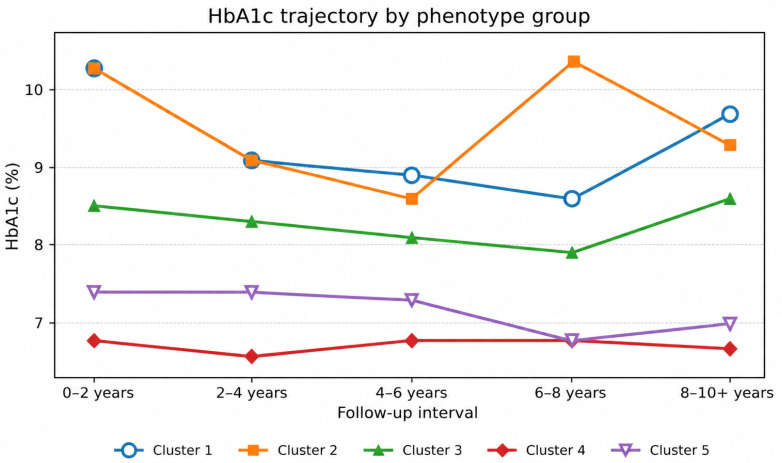
Median HbA1c values during predefined follow-up intervals. The number of patients contributing data to each interval is provided in Table 4.

HbA1c values during follow-up were evaluated across predefined post-diagnosis intervals: 0–2 years, 2–4 years, 4–6 years, 6–8 years, and 8–10+ years. The number of available observations decreased over time because of the retrospective design and unequal follow-up duration. Descriptive HbA1c values according to phenotype group and time interval are presented in Table 4. The final interval was open-ended and included available observations beyond 10 years when present. Changes in median HbA1c values during follow-up across phenotype groups are shown in Figure 2.

## 4. Discussion

In this retrospective study, patients with diabetes were evaluated according to five previously described cluster-derived phenotype groups using baseline clinical and biochemical domains. The findings suggest that these groups differ in baseline metabolic characteristics and in documented complication profiles during follow-up. Because no new unsupervised clustering algorithm was performed and because follow-up data were incomplete, the results should be interpreted as exploratory associations rather than evidence of individual-level prediction, causal inference, or clinical decision-making.

Cluster 1 was consistent with the autoimmune diabetes phenotype, Cluster 2 with marked insulin deficiency, and Cluster 3 with insulin resistance and obesity. The inclusion of type 1 diabetes and LADA cases in Cluster 1 supports the autoimmune nature of this group. The findings that Cluster 1 was characterized by lower BMI, earlier age at onset, poor glycemic control, and a tendency toward ketosis; Cluster 2 by beta-cell deficiency and higher HbA1c levels; and Cluster 3 by higher BMI and HOMA-IR values are consistent with phenotypic characteristics reported in the literature [9,10,11,12].

C-peptide and HOMA1-%B-related variables were used to represent beta-cell function. Lower C-peptide and beta-cell function indices were observed in insulin-deficient phenotype groups, whereas higher C-peptide and HOMA1-%B values were observed in insulin-resistant or obesity-related groups. This pattern is compatible with the clinical logic of the Ahlqvist/Zaharia framework, although the present study should not be interpreted as an independent validation of a new clustering algorithm.

HOMA-IR and BMI category were used as key variables to identify insulin resistance and obesity-related phenotypes. Obesity was concentrated in Clusters 3 and 4 by definition, and HOMA-IR was numerically higher in insulin resistance-related groups, although the continuous HOMA-IR comparison was not statistically significant in the available-case analysis. These findings support the clinical logic of the phenotype assignment structure but should not be interpreted as independent discovery of new clusters.

When HbA1c levels were evaluated, metabolic control was poorer in Clusters 1 and 2, whereas HbA1c values were lower in Clusters 4 and 5. The longitudinal mixed-effects model showed a significant overall group effect but did not show a significant group-by-time interaction. Therefore, HbA1c findings should be interpreted as differences in documented glycemic course, not as evidence of differential therapeutic effects.

In terms of lipid parameters, HDL cholesterol differed significantly among groups in the continuous-variable analysis, whereas triglyceride, total cholesterol, and LDL cholesterol did not. The lower HDL profile in Cluster 3 remains compatible with an insulin resistance-related metabolic phenotype [13].

Hepatic steatosis/suspected NAFLD was more frequent in Clusters 3 and 4. This finding is consistent with the association between insulin resistance, obesity, and hepatic fat accumulation. However, because most diagnoses were based on routine clinical imaging reports, the term suspected NAFLD is more appropriate than NASH unless histological or validated noninvasive diagnostic criteria are documented.

When diabetic complications were evaluated, retinopathy, nephropathy, and polyneuropathy showed different distributions among phenotype groups. Retinopathy was more prominent in Cluster 2, which may be related to poor glycemic control and beta-cell deficiency. Previous studies have also reported that glycemic control is a key determinant of diabetic retinopathy development [14,15]. Nephropathy was most frequently observed in Cluster 3, suggesting that renal complication risk may be higher in phenotypes associated with insulin resistance and obesity [16]. Polyneuropathy was also more frequent in Cluster 2, suggesting that neuropathic complications may be more common in phenotypes characterized by insulin deficiency and poor metabolic control [17,18].

In exploratory phenotype-contrast analysis, the association between Clusters 2–3 and coronary artery disease did not reach statistical significance. Cerebrovascular events were rare and were not modeled because only three events were documented. These findings should be interpreted cautiously because of the small number of macrovascular events and the retrospective design.

Ketosis and ketoacidosis were most frequently observed in insulin-deficient phenotype groups. In the exploratory contrast analysis, Clusters 1–2 had higher odds of ketosis/ketoacidosis than the remaining groups after adjustment for current age, sex, and diabetes-duration category. This finding is compatible with the concept that reduced insulin secretion capacity may increase the risk of metabolic decompensation, but it should be interpreted as an exploratory association rather than as evidence of individual-level prediction.

Overall, the findings indicate that cluster-derived phenotype groups may differ in glycemic profiles and documented complication patterns. Poor glycemic control and ketosis/ketoacidosis were more common in insulin-deficient groups, whereas hepatic steatosis/suspected NAFLD and nephropathy were more common in insulin-resistant or obesity-related groups. These results may help describe clinical heterogeneity in diabetes, but they should not be used for individual-level prediction or clinical decision-making.

Several limitations should be acknowledged. This was a single-center retrospective study with a modest sample size and incomplete follow-up data. Outcome ascertainment was based on routine clinical records rather than standardized prospective screening, and reliable distinction between prevalent and incident complications was not possible for all outcomes. Residual confounding may remain because some variables, including smoking status, blood pressure, treatment regimen, treatment adherence, therapeutic intensification, and follow-up intensity, were not consistently available in a standardized analyzable format. In addition, no new unsupervised clustering algorithm or external validation was performed, and the phenotype-contrast regression analyses were exploratory and post hoc.

## 5. Conclusions

This study showed that patients classified according to cluster-derived diabetes phenotype groups had different baseline clinical and metabolic characteristics and different documented complication profiles. Exploratory phenotype-contrast regression analyses suggested that selected phenotype contrasts were associated with specific documented complications, but these results should be interpreted cautiously because the contrasts were post hoc and no formal correction for multiple testing was applied. HbA1c levels also differed between groups during follow-up, although the longitudinal model did not show a significant time effect or group-by-time interaction. These findings support the descriptive value of phenotype-based classification for understanding clinical heterogeneity in diabetes. Because of the retrospective design, incomplete data, lack of a new unsupervised clustering procedure, and exploratory nature of the regression analyses, the results should be considered hypothesis-generating and should be confirmed in larger prospective cohorts.

## Figures and Tables

**Table 1:** Baseline characteristics according to diabetes phenotype group.

Variable	Total	Cluster 1 (n = 36)	Cluster 2 (n = 29)	Cluster 3 (n = 35)	Cluster 4 (n = 26)	Cluster 5 (n = 32)	*p* Value
Age, years	52.0 (43.2–64.0)	40.0 (29.5–44.0)	53.0 (47.0–63.0)	53.0 (45.5–65.5)	59.0 (51.2–63.8)	64.0 (51.8–70.2)	<0.001
Female sex, n/N (%)	68/158 (43.0)	10/36 (27.8)	11/29 (37.9)	18/35 (51.4)	17/26 (65.4)	12/32 (37.5)	0.034
Age at diagnosis, years	44.0 (34.0–53.0)	28.0 (16.0–39.5)	45.0 (36.0–50.0)	40.0 (35.0–51.5)	51.0 (45.0–54.0)	53.0 (45.5–63.0)	<0.001
Diabetes duration, years	9.0 (5.0–14.0)	7.0 (2.0–15.5)	12.0 (7.0–15.0)	10.0 (7.0–14.0)	8.0 (6.0–12.0)	6.5 (4.0–11.2)	0.090
Baseline HbA1c, %	8.4 (6.9–10.3)	10.3 (8.9–11.0)	10.3 (8.1–12.2)	8.5 (6.9–10.3)	6.8 (6.3–7.1)	7.4 (6.6–8.4)	<0.001
Obesity (BMI ≥ 30 kg/m^2^), n/N (%)	63/158 (39.9)	2/36 (5.6)	0/29 (0.0)	35/35 (100.0)	26/26 (100.0)	0/32 (0.0)	<0.001
Autoantibody positivity, n/N (%)	29/158 (18.4)	29/36 (80.6)	0/29 (0.0)	0/35 (0.0)	0/26 (0.0)	0/32 (0.0)	<0.001
Fasting plasma glucose (mg/dL)	168 (133–276)	200 (157–313)	281 (176–330)	140 (122–152)	128 (122–133)	183 (137–232)	<0.001
C-peptide (ng/mL)	1.05 (0.25–2.19)	0.17 (0.10–0.38)	1.15 (0.72–1.92)	3.48 (2.52–4.81)	3.28 (2.23–3.42)	1.68 (1.07–1.93)	<0.001
Fasting insulin (µIU/mL)	11.56 (4.19–22.67)	1.59 (0.62–3.03)	4.29 (3.09–10.76)	22.97 (15.34–30.71)	13.69 (10.26–23.79)	8.56 (4.19–13.43)	0.004
HOMA-IR	5.30 (1.54–9.68)	0.69 (0.54–2.20)	1.54 (1.23–7.25)	7.17 (5.30–12.74)	4.02 (3.27–8.19)	3.61 (1.52–7.66)	0.143
HOMA1-%B, %	48.2 (17.8–98.1)	4.7 (0.8–5.0)	17.8 (9.2–21.5)	99.1 (71.7–168.8)	88.0 (56.2–114.1)	28.8 (11.6–52.0)	<0.001
Triglycerides (mg/dL)	162 (100–242)	130 (79–206)	171 (104–296)	193 (171–322)	156 (125–172)	156 (129–265)	0.138
Totalcholesterol (mg/dL)	194 (162–232)	178 (144–216)	191 (159–223)	205 (167–249)	210 (192–231)	200 (166–244)	0.420
LDLcholesterol (mg/dL)	112 (92–141)	104 (88–124)	114 (88–138)	112 (88–126)	132 (114–142)	110 (88–153)	0.411
HDLcholesterol (mg/dL)	45 (38–54)	48 (39–58)	41 (38–48)	40 (35–45)	52 (46–58)	44 (38–58)	0.040
ALT (U/L)	19 (14–30)	16 (11–21)	22 (16–28)	29 (22–36)	18 (16–28)	20 (13–30)	0.049
AST (U/L)	19 (16–24)	18 (14–23)	20 (14–25)	21 (17–24)	18 (18–23)	18 (15–20)	0.539
Creatinine (mg/dL)	0.80 (0.70–0.90)	0.80 (0.60–0.90)	0.80 (0.70–0.90)	0.80 (0.60–0.93)	0.80 (0.70–0.90)	0.90 (0.80–1.00)	0.758
eGFR (mL/min/1.73 m^2^)	99 (85–111)	115 (111–118)	100 (99–108)	106 (96–108)	92 (78–100)	86 (85–96)	0.017

Values are median (interquartile range) unless otherwise stated. Obesity was based on available categorical BMI data. HOMA1-%B was calculated using the classical HOMA1 formula and should not be interpreted as Oxford HOMA2-%B. Analyses used available cases.

**Table 2:** Binary complication outcomes according to diabetes phenotype group.

Outcome	Cluster 1 (n/N, %)	Cluster 2 (n/N, %)	Cluster 3 (n/N, %)	Cluster 4 (n/N, %)	Cluster 5 (n/N, %)	*p* Value	Cramer’s V
Hepatic steatosis/ suspected NAFLD	7/36 (19.4)	11/29 (37.9)	35/35 (100.0)	23/26 (88.5)	12/32 (37.5)	<0.001	0.648
Diabetic retinopathy	9/36 (25.0)	18/29 (62.1)	13/35 (37.1)	4/26 (15.4)	7/32 (21.9)	0.001	0.338
Diabetic nephropathy	10/36 (27.8)	16/29 (55.2)	27/35 (77.1)	2/26 (7.7)	7/32 (21.9)	<0.001	0.510
Diabetic polyneuropathy	14/36 (38.9)	19/29 (65.5)	16/34 (47.1)	7/26 (26.9)	7/32 (21.9)	0.005	0.307
Coronary artery disease	3/36 (8.3)	9/29 (31.0)	10/35 (28.6)	3/26 (11.5)	9/32 (28.1)	0.070	0.234
Cerebrovascular event	0/36 (0.0)	2/29 (6.9)	1/35 (2.9)	0/26 (0.0)	0/32 (0.0)	0.208	0.193
Ketosis/ketoacidosis	20/36 (55.6)	23/29 (79.3)	20/35 (57.1)	4/26 (15.4)	3/32 (9.4)	<0.001	0.523

Values are positive cases/available denominator (%). For each outcome, values coded 1–5 in the source dataset were classified as ever-positive, and 0 was classified as negative.

**Table 3:** Exploratory phenotype-contrast logistic regression analyses for selected binary outcomes. Models were adjusted for current age, sex, and diabetes-duration category. Baseline HbA1c was not included because HbA1c at diagnosis was used for phenotype assignment. Contrasts were selected post hoc according to the observed complication distribution and clinical phenotype structure; no formal correction for multiple testing was applied.

Outcome	Exploratory Phenotype Contrast	Model N/Events	Event Frequency in Contrast vs. Reference	Adjusted OR (95% CI)	*p* Value
Hepatic steatosis/ suspected NAFLD	Clusters 3–4 vs. others	158/88	58/61 vs. 30/97	39.84 (11.13–142.67)	<0.001
Diabetic retinopathy	Cluster 2 vs. others	158/51	18/29 vs. 33/129	4.51 (1.84–11.07)	0.001
Diabetic nephropathy	Cluster 3 vs. others	158/62	27/35 vs. 35/123	7.67 (3.15–18.70)	<0.001
Diabetic polyneuropathy	Cluster 2 vs. others	157/63	19/29 vs. 44/128	3.52 (1.41–8.77)	0.007
Coronary artery disease	Clusters 2–3 vs. others	158/34	19/64 vs. 15/94	2.08 (0.92–4.71)	0.078
Ketosis/ketoacidosis	Clusters 1–2 vs. others	158/70	43/65 vs. 27/93	4.15 (1.94–8.88)	<0.001

**Table 4:** HbA1c follow-up values by phenotype group and follow-up interval.

Group	0–2 Years	2–4 Years	4–6 Years	6–8 Years	8–10+ Years
Total	148; 8.4 (6.9–10.3)	134; 7.7 (6.7–9.5)	122; 7.7 (6.8–9.0)	96; 7.9 (6.9–10.1)	70; 8.6 (7.0–9.9)
Cluster 1	31; 10.3 (8.9–11.0)	23; 9.1 (7.6–10.3)	25; 8.9 (7.6–10.1)	17; 8.6 (8.2–10.7)	19; 9.7 (8.7–10.2)
Cluster 2	28; 10.3 (8.1–12.2)	28; 9.1 (7.2–11.3)	22; 8.6 (7.3–10.4)	19; 10.4 (8.4–12.2)	15; 9.3 (8.2–11.1)
Cluster 3	34; 8.5 (6.9–10.3)	30; 8.3 (7.2–9.4)	28; 8.1 (6.8–9.0)	26; 7.9 (7.5–9.6)	15; 8.6 (7.3–9.4)
Cluster 4	24; 6.8 (6.3–7.1)	22; 6.6 (6.2–7.2)	21; 6.8 (6.6–7.4)	14; 6.8 (6.3–7.5)	10; 6.7 (6.5–7.0)
Cluster 5	31; 7.4 (6.6–8.4)	31; 7.4 (6.3–8.0)	26; 7.3 (6.3–8.3)	20; 6.8 (6.2–7.9)	11; 7.0 (6.5–7.6)

Cells show n; median (interquartile range). HbA1c measurements were summarized within predefined post-diagnosis intervals. The final interval was open-ended and included available HbA1c observations from 8 years onward, including observations beyond 10 years when present. Linear mixed-effects model results: phenotype group *p* < 0.001, follow-up interval *p* = 0.292, and group-by-time interaction *p* = 0.293.

## Data Availability

The data that support the findings of this study are not publicly available due to privacy and ethical restrictions.

## Abbreviations

The following abbreviations are used in this manuscript:

BMI	Body mass index
CI	Confidence interval
DM	Diabetes mellitus
GADA	Glutamic acid decarboxylase antibody
HbA1c	Glycated hemoglobin
HDL	High-density lipoprotein
HOMA-IR	Homeostatic Model Assessment of Insulin Resistance
LADA	Latent autoimmune diabetes in adults
LDL	Low-density lipoprotein
MARD	Mild age-related diabetes
NAFLD	Non-alcoholic fatty liver disease
MOD	Mild obesity-related diabetes
NASH	Non-alcoholic steatohepatitis
OR	Odds ratio
SAID	Severe autoimmune diabetes
SIDD	Severe insulin-deficient diabetes
SIRD	Severe insulin-resistant diabetes
